# A pH-Sensitive Sprayable Fluorescent Probe Enables Accurate Visualization of Thyroid Cancer Margins for Fluorescence-Guided Surgery in Orthotopic Mouse Models

**DOI:** 10.3390/cancers18040632

**Published:** 2026-02-15

**Authors:** Hyungju Kwon, Javier Bravo, Ting-Chun Kuo, Blackberrie Eddins, Siamak Amirfakhri, Jasmin Zaker, Keita Kobayashi, Shinya Yokomizo, Homan Kang, Grace Lin, Md Shamim, Hak Soo Choi, Robert M. Hoffman, Satoshi Kashiwagi, Maged Henary, Michael Bouvet

**Affiliations:** 1 Department of Surgery, University of California San Diego, San Diego, CA 92092, USA; hkwon@ewha.ac.kr (H.K.); jbquintana@health.ucsd.edu (J.B.); tinakuo1204@gmail.com (T.-C.K.); beddins@health.ucsd.edu (B.E.); siamirfakhri@health.ucsd.edu (S.A.); zakerjasmin@gmail.com (J.Z.); kjkobayashi@ucsd.edu (K.K.); rhoffman@health.ucsd.edu (R.M.H.); 2 Department of Surgery, Ewha Womans University College of Medicine, Seoul 07985, Republic of Korea; 3 VA San Diego Healthcare System, San Diego, CA 92161, USA; 4 Department of Surgery, National Taiwan University Hospital, Taipei 100225, Taiwan; 5 Center for Inflammation Imaging, Department of Radiology, Massachusetts General Hospital and Harvard Medical School, Boston, MA 02114, USA; syokomizo@mgh.harvard.edu (S.Y.); hkang7@mgh.harvard.edu (H.K.); skashiwagi@mgh.harvard.edu (S.K.); 6 Department of Pathology, University of California San Diego, San Diego, CA 92092, USA; g4lin@health.ucsd.edu; 7 Department of Chemistry, Center for Diagnostics and Therapeutics, Georgia State University, Atlanta, GA 30302, USA; mdshamim4200@gmail.com (M.S.); mhenary1@gsu.edu (M.H.); 8 Gordon Center for Medical Imaging, Department of Radiology, Massachusetts General Hospital and Harvard Medical School, Boston, MA 02114, USA; hchoi12@mgh.harvard.edu; 9 AntiCancer Inc., San Diego, CA 92111, USA

**Keywords:** fluorescence-guided surgery, thyroid carcinoma, near-infrared imaging, tumor margin detection, orthotopic mouse model

## Abstract

Positive surgical margins (PSMs) are a major predictor of recurrence in thyroid cancer; however, their intraoperative detection remains challenging, particularly for microscopic PSMs. This study aimed to demonstrate that a sprayable pH-sensitive near-infrared fluorescent probe (PH10) could specifically and robustly label thyroid tumors in orthotopic mouse models. The pH sensitivity, cytotoxicity, and cellular uptake mechanisms of PH10 were evaluated in papillary thyroid carcinoma (K1) and anaplastic thyroid carcinoma (8505C) cell lines in vitro. Orthotopic thyroid cancer mouse models were then established using both K1 and 8505C cells. In vivo fluorescence following topical spraying of PH10 was quantified after sequential washes to assess tumor-to-background ratios. Fluorescence-guided surgery (FGS) was performed to determine whether PH10 could facilitate complete resection of orthotopically grown thyroid tumors. Complete resection was validated by hematoxylin and eosin histology. PH10 demonstrated low cytotoxicity at clinically relevant concentrations in vitro and showed selective uptake into thyroid cancer cells, predominantly via organic anion transporting polypeptide-mediated transport. Topical spraying of PH10 in orthotopic thyroid cancer mouse models established from K1 and 8505C cell lines generated strong tumor-specific fluorescence. Tumor-to-background ratios were significantly higher than background and remained distinguishable after multiple washes. In both models, PH10 enabled visualization of macroscopic residual tumors and detection of microscopic PSMs, with fluorescence patterns closely corresponding to histologic findings. FGS enabled complete tumor resection, as confirmed by fluorescence and histology. In conclusion, topical application of PH10 provides rapid, tumor-specific fluorescence suitable for identifying PSMs and facilitating complete tumor resection by FGS in thyroid cancer.

## Simple Summary

Surgery remains the cornerstone of treatment for thyroid carcinoma. However, intraoperative identification of tumor margins remains challenging and can hinder complete tumor removal. In this study, we applied a pH-sensitive sprayable fluorescent probe to detect thyroid cancer and guide resection in orthotopic nude mouse models. The fluorophore generated bright, highly specific fluorescence signals that facilitated complete tumor resection in these models. Overall, these results highlight the potential of this pH-sensitive probe to improve surgical precision in thyroid cancer.

## 1. Introduction

Thyroid cancer is the third most common malignancy in women, with an estimated 825,890 affected female patients in the United States as of 1 January 2025 [1]. Although most thyroid cancers are associated with an excellent prognosis, approximately 20–30% demonstrate clinically aggressive behavior, including locoregional recurrence or distant metastasis [2]. Previous studies have sought to identify patients at high-risk compared with those with a more favorable clinical course [3,4]. Clinical and pathological variables—such as age, sex, primary tumor size, multifocality, or surgical margin status—have been widely explored as potential predictors of recurrence [5,6]. Among these, the prognostic relevance of surgical margin involvement has garnered growing interest.

Positive margins indicate residual tumor cells at the resection border and are a major determinant of postoperative recurrence and decreased survival [7,8]. However, intraoperative identification of margin involvement is challenging, particularly in cases of microscopic positive surgical margins (mPSMs). mPSMs constitute the majority of positive margins, and frozen-section examination is typically used to detect them in suspicious cases [9]. Conventional frozen-section analysis, however, is time-consuming, subject to sampling errors, and often unreliable in thyroid cancer due to misinterpretation [10]. Consequently, surgical decision-making still largely relies on visual inspection under bright light and palpation, which are often insufficient for accurate margin assessment. These limitations underscore the importance of real-time, sensitive, and tumor-specific techniques to visualize residual tumor tissue during surgery.

Fluorescence-guided surgery (FGS) has recently emerged as a promising tool for intraoperative tumor visualization [11]. FGS has improved tumor detection and resection accuracy in colorectal cancer, brain tumors, and breast cancer [12,13]. A recent meta-analysis demonstrated that near-infrared (NIR) imaging shows promise for real-time surgical margin assessment in head and neck cancer [14]. Spray-type fluorophores offer several advantages, such as rapid generation of tumor-specific contrast, lower false-negative rates, and reduced systemic toxicity, compared with conventional intravenous delivery methods [15,16]. Our previous studies have also indicated that sprayable pH-sensitive NIR probes effectively delineate malignant thyroid tumors, enabling complete resection while preserving adjacent normal structures [11].

In the present study, we aimed to show that the sprayable pH-sensitive fluorescent probe (PH10) could label residual thyroid tumors with bright fluorescence in orthotopic mouse models, thereby enabling complete tumor resection by FGS.

## 2. Materials and Methods

### 2.1. Study Design

We initially verified the pH responsiveness and in vitro cellular uptake of the pH-sensitive probe PH10 in K1 thyroid carcinoma cells (Supplementary Figure S1). Subsequently, we conducted in vivo studies to evaluate whether PH10 could selectively label residual thyroid carcinoma in an orthotopic mouse model. Next, we assessed its ability to detect residual tumors in the tumor bed after resection. Finally, we validated these findings using 8505C, a more aggressive thyroid cancer cell line. Because anaplastic thyroid carcinoma (ATC) is characterized by an aggressive phenotype and extensive infiltration into surrounding tissues, margin involvement is more common and clinically challenging in patients with this type of cancer. Thus, we selected 8505C cells as a model for highly aggressive thyroid cancers. All procedures were conducted under protocols approved by the UCSD Institutional Animal Care and Use Committee (protocol code S99001 and date of approval 22 January 2025).

### 2.2. Cell Culture

The human papillary thyroid carcinoma (PTC) cell line K1 and the human ATC cell line 8505C were purchased from the ATCC (American Type Culture Collection; Manassas, VA, USA). Cells were maintained in Dulbecco’s Modified Eagle Medium supplemented with 10% fetal bovine serum (FBS) (Gibco, Grand Island, NY, USA), 1% insulin-transferrin-selenium (Sigma-Aldrich, St. Louis, MO, USA), and 1% penicillin-streptomycin (Gibco) or RPMI1640 supplemented with 10% FBS, 1% penicillin-streptomycin in a humidified 5% CO_2_ atmosphere. Cells were routinely passaged to maintain viability and proliferation.

### 2.3. Orthotopic Mouse Models

Thyroid carcinoma mouse models were established using the surgical orthotopic implantation (SOI) method as previously described [11]. In brief, 4–6 weeks-old athymic nu/nu nude mice (both sexes) were obtained from The Jackson Laboratory (Bar Harbor, ME, USA) and maintained in the institutional animal facility with access to sterilized food and water. Prior to surgical procedures, mice were anesthetized using 200–300 μL of a ketamine cocktail composed of 1 mL ketamine, 0.1 mL xylazine, and 8.9 mL of phosphate-buffered saline (PBS). For postoperative analgesia, 150 μL of buprenorphine (0.05 mg/kg) diluted in PBS was administered subcutaneously. After anesthesia, the neck was disinfected with 70% ethanol, and a small incision was made. The salivary glands were gently retracted to expose the trachea and identify the thyroid gland. Either K1 cells (1 × 10^6^ cells) or 8505C cells (1 × 10^6^ cells) were then injected into the thyroid gland using a 30-gauge needle. Following cell injection, the salivary glands were repositioned and the incision was closed with 6-0 sutures. Mice were monitored postoperatively, and tumors typically became palpable after approximately 4 weeks.

### 2.4. Cytotoxicity Assay for PH10

We assessed the cytotoxicity of the pH-sensitive probe using the Cell Counting Kit-8 (CCK-8) from Dojindo Molecular Technologies, Inc. (Kumamoto, Japan). K1 or 8505C cells were cultured in 96-well plates at a density of 3000 cells per well and treated with 0 to 50 μM of PH10 in growth media for 24 h. We then added 10% CCK-8 solution to each well. We incubated the plates at 37 °C for an additional 4 h and measured absorbance at 450 nm using a Cytation5 (BioTek, Winooski, VT, USA). Cell viability was determined by comparing the absorbance of the sample to that of the blank and negative control wells. We evaluated the IC_50_ (half-maximal inhibitory concentration) values from the CCK-8 assay using nonlinear regression with sigmoidal concentration–response curves in GraphPad Prism 10 (GraphPad Software Inc., Boston, MA, USA).

### 2.5. Cellular Uptake Inhibition Assay

To investigate the role of organic anion transporter polypeptides (OATPs) in PH10 uptake by thyroid cancer cells, we pretreated K1 or 8505C cells with 250 μM of bromsulphthalein (BSP) (Sigma-Aldrich) or 10 μM of MK-571 (Cayman Chemical, Ann Arbor, MI, USA) for 5 min, followed by incubation with 0.2 μM of PH10 at 37 °C for 15 min. To assess membrane diffusion, we incubated separate wells at 4 °C. After washing, fluorescence images were acquired using a Cytation5. Phase-contrast images were acquired to delineate cell outlines; the resulting regions of interest were overlaid on fluorescence images for quantification. Fluorescence intensity per cell was measured using ImageJ, version 1.54r (National Institutes of Health, Bethesda, MD, USA) and CellProfiler version 4.2.8 (Broad Institute, Cambridge, MA, USA) [17].

### 2.6. Sprayable PH10-Solution Preparation

A 5 weight/volume% bovine serum albumin (BSA) solution was made by dissolving 0.5 g of BSA in 10 mL of saline. To prepare a 10 μM working solution of PH10, a 10 mM PH10 stock solution in dimethyl sulfoxide (DMSO) was diluted with 5% BSA. For tumor spraying, 200 μL of this working solution was applied to each mouse.

### 2.7. Spraying PH10 on Orthotopic Thyroid Cancers in Nude Mice

For sprayable PH10 application on thyroid cancers, mice bearing orthotopic cancers were euthanized via CO_2_ inhalation followed by cervical dislocation. The overlying skin was removed, and surrounding tissues including muscle, and salivary glands were carefully dissected away to expose the tumor and adjacent normal tissues without causing injury. The PH10 working solution was then loaded into a syringe and sprayed onto the exposed cancer and surrounding area. Bright-field and NIR images were obtained immediately after spraying and after three sequential washes with PBS.

### 2.8. NIR Fluorescence Imaging

NIR imaging was performed using the Pearl Trilogy Small Animal Imaging System (LI-COR Biosciences, Lincoln, NE, USA). Bright-field images were acquired using the white light channel, while NIR images were collected through the 800 channel immediately after probe application and following each wash. After imaging, tumors, peritumoral regions, and adjacent tissues were harvested for histologic evaluation. Regions of interest were defined based on the fluorescence images, and mean fluorescence intensities (mFI) for tumor and background regions were measured using the Pearl Imager version 5.2. Tumor-to-background ratios (TBRs) were then calculated by dividing tumor mFI by background mFI to assess probe retention and specificity after spraying and subsequent PBS washes.

### 2.9. Hematoxylin and Eosin (H&E) Staining

Orthotopic K1 and 8505C tumors, along with adjacent tissues, were harvested intact and fixed in formalin for at least 72 h before paraffin embedding. The paraffin blocks were sectioned, and the resulting tissue sections were stained with H&E following standard procedures.

### 2.10. Definition of Margin Involvement

Margin involvement is defined as the presence of cancer cells at the external resection surface and can be classified as macroscopic or microscopic. A macroscopic positive margin refers to overt residual tumor visible on the operative bed. In contrast, a mPSM is defined as the absence of gross residual tumor, with cancer cells identified only on histopathologic examination using H&E staining.

### 2.11. Statistical Analysis

For the cellular uptake study, we used a one-way ANOVA (analysis of variance) following by Tukey’s multiple comparison test in GraphPad Prism 10. Fluorescence data were analyzed using R, version 4.4 (R Foundation for Statistical Computing, Vienna, Austria). Normality of the data distribution was assessed using the Shapiro–Wilk test. For repeated measurements across time points (after spraying and subsequent washes), the Friedman test was applied, followed by pairwise Wilcoxon signed-rank tests to examine differences between specific stages. For post-resection comparisons, mFI values of excised tumors and the corresponding tumor beds were analyzed using a two-tailed paired *t* test on log-transformed data to evaluate mean differences. Geometric mean ratios and 95% confidence intervals (CIs) were obtained by exponentiating the mean log differences. A *p* value < 0.05 was considered statistically significant.

## 3. Results

### 3.1. Cytotoxicity of PH10

**Figure 1:**
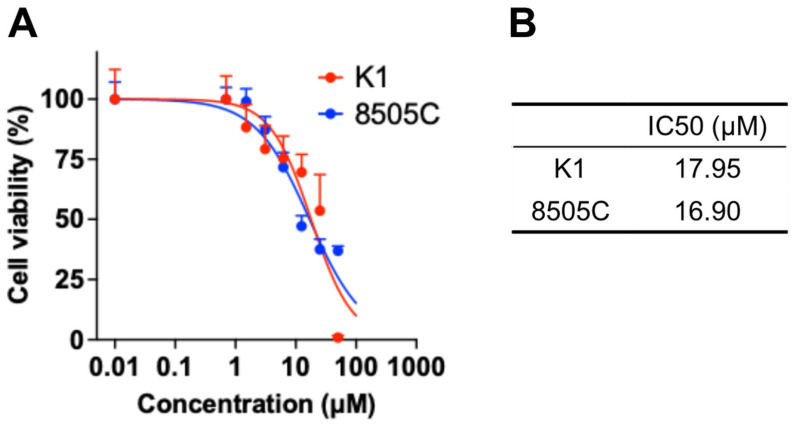
Cytotoxicity profile of PH10 in thyroid cancer cell lines. (**A**) Dose–response curves showing cell viability of K1 and 8505C cells after exposure to increasing concentration of PH10. (**B**) Calculated half-maximal inhibitory concentration (IC_50_) values of PH10 for K1 and 8505C cells.

We assessed the cytotoxic potential of PH10 in thyroid cancer cell lines at probe concentrations ranging from 0 to 50 µM (Figure 1). The IC_50_ value of PH10 were 17.95 µM and 16.90 µM in K1 and 8505C cells, respectively. These results support that topical administration of PH10 at 10 µM, as used in this study, is unlikely to be cytotoxic under the conditions tested.

### 3.2. Thyroid Cancer Cell Targetability of PH10

**Figure 2:**
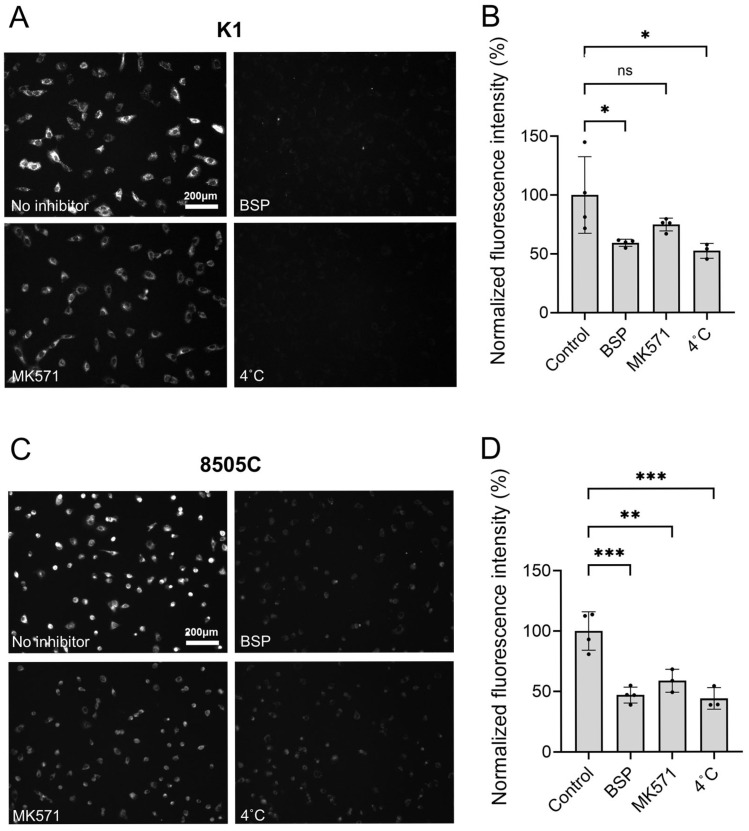
OATP-dependent cellular uptake of PH10 in papillary and anaplastic thyroid cancer cells. Representative fluorescence images of (**A**) K1 and (**C**) 8505C cells. Quantitative fluorescence intensity measurements in (**B**) K1 and (**D**) 8505C cells. ns, not significant, * *p* < 0.05, ** *p* < 0.01, *** *p* < 0.001.

Our previous study showed that pH-sensitive heptamethine cyanine analogs, including PH10, were predominantly taken up via OATP [11,18]. To confirm this in K1 and 8505C cells, PH10 uptake was assessed in the presence of OATP inhibitors, BSP or MK-571, and cells were also incubated at 4 °C to suppress membrane transporter activity (Figure 2A). In K1 cells, PH10 fluorescence intensity was markedly decreased after incubation with BSP or at 4 °C (*p* < 0.05), compared to the control (Figure 2B). MK-571 also reduced PH10 fluorescence intensity. Together, these results indicate that PH10 uptake is predominantly mediated by OATP (Figure 2A). Consistently, the PH10 signal was significantly reduced in 8505C cells treated with BSP, MK-571, or incubated at 4 °C, compared to the control (Figure 2C,D).

### 3.3. PH10 Spray Labeling of K1 Thyroid Tumors in an Orthotopic Mouse Model

**Figure 3:**
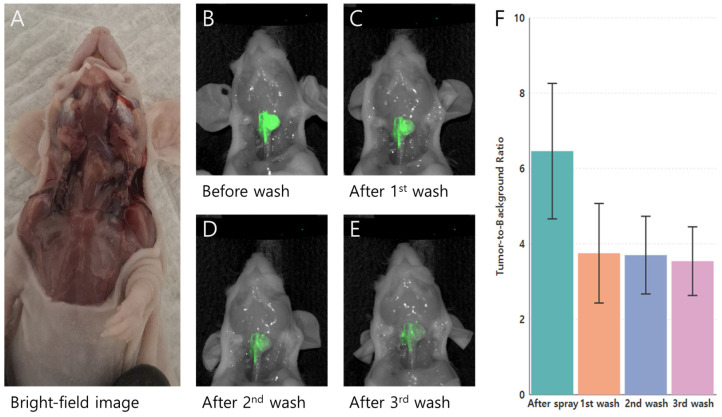
Wash-resistant fluorescence of PH10 in an orthotopic K1 thyroid carcinoma mouse model (*n* = 5) following topical application. (**A**) Bright-field image showing the exposed cervical region and orthotopic thyroid tumor prior to fluorescence imaging; (**B**–**E**) Near-infrared fluorescence images acquired immediately after topical spraying of PH10 and after sequential PBS washes, demonstrating persistent tumor-specific fluorescence despite repeated washing; (**F**) Quantitative analysis of tumor-to-background ratios (TBRs) before washing and after each sequential wash.

The tumor-targeting and labeling capabilities of PH10 were assessed in orthotopic nude mouse models (*n* = 5) using the human PTC cell line K1. Topical application of PH10 at 10 μM produced strong tumor fluorescence immediately after spraying, which progressively decreased following sequential washes with PBS (Figure 3). The TBR was highest immediately after spraying (6.46 ± 1.80) and declined after the first wash (3.75 ± 1.32), with smaller incremental decreases after the second (3.70 ± 1.03) and third (3.54 ± 0.91) washes. Overall differences in TBR over time were statistically significant based on the Friedman test (*p* = 0.026), indicating a wash-dependent change in fluorescence. Pairwise comparisons did not reach statistical significance for individual time point contrasts. However, all comparisons involving the post-spraying condition demonstrated consistent downward trends (*p* ≈ 0.06), reflecting a rapid reduction in nonspecific fluorescence after the initial wash. No meaningful differences were observed between later wash steps, suggesting relative stabilization of the tumor-associated signal following early clearance. These findings indicate that serial washing effectively reduces background fluorescence in K1 tumors, with the greatest impact after the first wash; the fluorescence signal subsequently stabilized.

### 3.4. Multistep FGS to Completely Resect the Orthotopic K1 Thyroid Tumors After PH10 Spray Labeling

**Figure 4:**
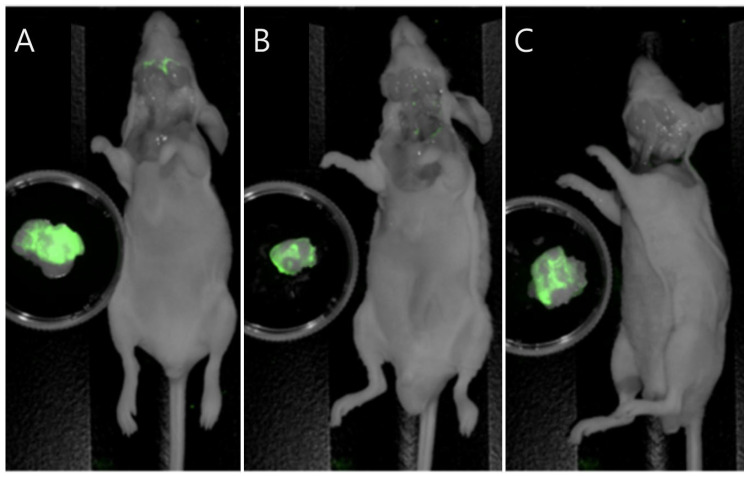
Fluorescence-guided detection of residual thyroid tumor following total thyroidectomy in an orthotopic K1 thyroid cancer mouse model (*n* = 5). (**A**–**C**) Representative near-infrared fluorescence images of orthotopic K1 thyroid cancer mouse models following total thyroidectomy with excised tumors positioned adjacent to the corresponding mice to assess residual fluorescence after resection. Tumors retain strong PH10 fluorescence, whereas the tumor beds exhibit minimal signal.

The efficacy of PH10 for positive margin detection was further evaluated in the orthotopic K1 thyroid cancer mouse models. Total thyroidectomy procedures for tumor resection were performed in mice. Excised tumors demonstrated substantially higher fluorescence than the corresponding tumor beds, with a mean TBR of 3.42 ± 0.82. This difference was statistically significant (*p* = 0.003), indicating robust tumor-specific signal retention and minimal residual fluorescence within the resection bed (Figure 4).

**Figure 5:**
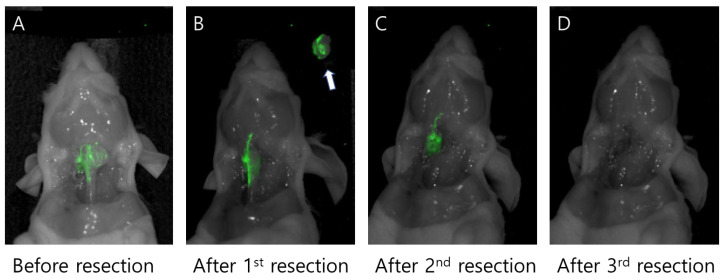
Serial removal of margins in an orthotopic K1 thyroid cancer mouse model. (**A**) before tumor resection; (**B**) after first resection, showing residual fluorescence in the trachea. The resected tumor is shown (white arrow); (**C**) after second resection, indicating remnant tumor after trachea removal; (**D**) after third resection, demonstrating complete tumor removal with no remnant tumor.

Following the first tumor resection, the margins were progressively widened until no fluorescence signal was detectable. These findings were compared with standard H&E histology. The average TBR values were 3.01 ± 0.51 for macroscopic residual tumors and 2.14 ± 0.41 for mPSMs. These TBRs enabled clear fluorescent delineation of the margins in all mice, allowing complete resection after three sequential resections (Figure 5).

### 3.5. Histopathologic Analysis of Orthotopic K1 Thyroid Cancers

**Figure 6:**
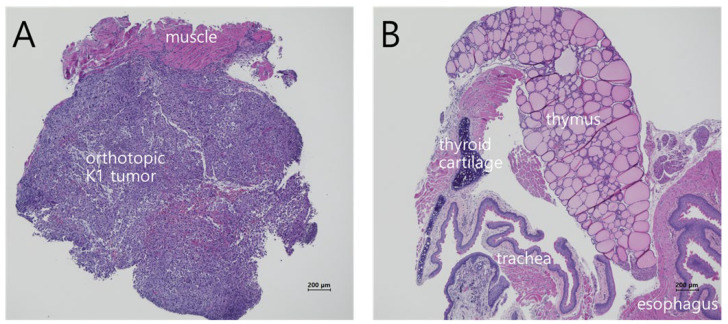
Histologic analysis of an orthotopic K1 thyroid carcinoma in nude mice. (**A**) Resection margin of the first-step FGS; (**B**) Operative bed showing no residual tumor after serial FGS.

K1 tumor sections were examined by H&E staining. These analyses confirmed a poorly differentiated thyroid cancer with invasion into adjacent skeletal muscle (Figure 6A). After serial margin widening, the operative bed showed no residual tumor, indicating a clear margin (Figure 6B).

### 3.6. Validation of PH10 Spray Labeling for Margin Detection in the Orthotopic 8505C ATC Mouse Model

**Figure 7:**
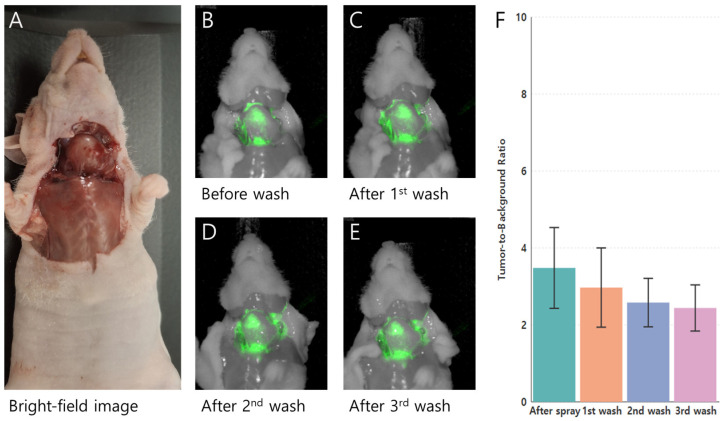
Orthotopic 8505C ATC nude mouse model (*n* = 10) for validation of PH10 for margin detection. (**A**) Bright-field image before PH10 spray; (**B**–**E**) Near-infrared fluorescence images of the tumor before and after PBS washes; (**F**) Mean tumor-to-background ratio (TBR) values.

Topical administration of 10 μM PH10 produced strong tumor fluorescence immediately after spraying in orthotopic 8505C ATC nude mouse models (*n* = 10). Fluorescence progressively decreased after sequential PBS washes (Figure 7). The mean TBR was 3.48 ± 1.05 immediately after spraying, 2.97 ± 1.03 after the first wash, 2.58 ± 0.63 after the second wash, and 2.44 ± 0.60 after the third wash. Pairwise comparisons between washes were not statistically significant (*p* > 0.05 for all), suggesting that nonspecific fluorescence was largely eliminated early in the wash sequence.

**Figure 8:**
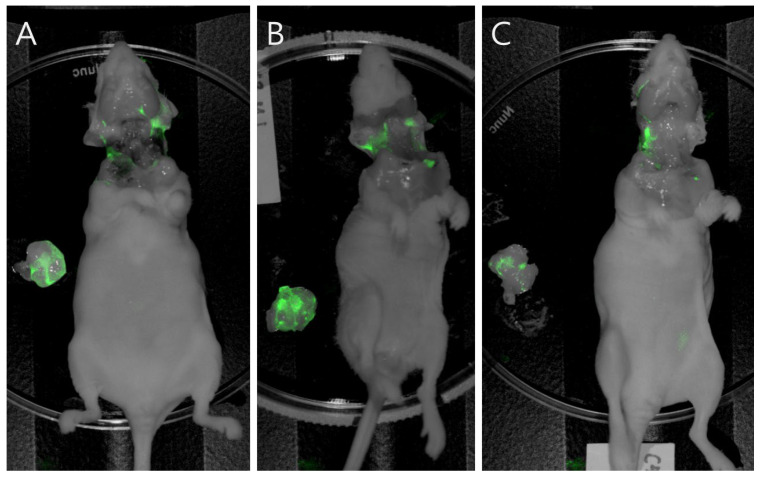
Fluorescence-guided identification of residual tumor and excised specimens following total thyroidectomy in an orthotopic 8505C thyroid cancer mouse model (*n* = 10). (**A**–**C**) Representative near-infrared fluorescence images from each mouse demonstrate that tumors retain strong PH10 fluorescence, whereas the resection bed shows minimal background signal.

For FGS of orthotopic 8505C carcinomas in mice sprayed with PH10, mean TBR values were 2.25 ± 0.24 for macroscopic residual tumor and 1.75 ± 0.36 for mPSM. These TBR values allowed clear fluorescence delineation of tumor margins in all mice. To further confirm tumor specificity, fluorescence intensities of excised tumors were compared with those of the corresponding tumor beds after resection (Figure 8). Tumor fluorescence remained significantly higher than background (*p* = 0.001), corresponding to a mean TBR of 2.27 ± 0.42 (95% CI 1.4–2.7). These findings demonstrate that PH10 selectively accumulates in tumor tissue and that residual fluorescence after washing reflects true tumor localization.

## 4. Discussion

The present study demonstrated that the sprayable pH-sensitive NIR fluorophore PH10 effectively labels residual thyroid carcinoma in orthotopic mouse models. PH10 exhibited strong affinity for thyroid cancer tissue and generated robust, tumor-specific fluorescence at tumor sites—features essential for FGS. Because the thyroid glands are closely associated with vital structures, including the trachea and esophagus, achieving complete tumor removal while preserving surrounding anatomy is particularly critical [19]. In the orthotopic mouse model, the TBR of residual cancer was significantly higher than that of normal tissue, allowing clear differentiation of surgical margins. Furthermore, our results indicate that PH10 can identify mPSM in thyroid cancer. Prior studies have similarly shown that pH-sensitive fluorophores can aid margin detection in several cancers, including liver, colon, and ovarian cancers, consistent with the present findings for thyroid cancer [16,20,21].

Interest is growing in the clinical implications of mPSM in thyroid cancer. A National Cancer Database analysis reported that 8.1% of patients with thyroid cancer had mPSM after resection [9]. Prior studies have reported that mPSM is associated with a nearly fivefold increased risk of recurrence [7,22]. Our recent work also found that mPSM exerted meaningful prognostic impact primarily in node-negative patients with PTC. Improvements in high-resolution ultrasonography have led to more frequent identification of early-stage thyroid cancers, resulting in a larger proportion of node-negative cases and underscoring the importance of mPSM in clinical practice [23]. Obtaining a clear margin remains critical in this large population, and the present technique can serve as a valuable tool to help achieve complete tumor resection.

Many researchers have attempted to identify margin involvement intraoperatively, with particular emphasis on detecting mPSM [24,25,26]. Various approaches have demonstrated encouraging potential, such as intraoperative ultrasonography, radiofrequency- or mass spectrometry-related devices, optical coherence tomography, and fluorescence-based margin evaluation [27]. Most of these techniques examine the excised specimen to determine margin status, while FGS visualizes the operative bed directly [28]. Evaluating margins solely on the resected specimens can be unreliable because electrosurgical instruments create thermal artifacts. Therefore, the present fluorescence-based strategy offers a distinct advantage in achieving more accurate margin assessment [29]. Consistent with this, our study showed that mice with a negative signal after resection had no residual tumor on histological analysis.

A sprayable probe for detecting mPSM is particularly advantageous in robotic (or endoscopic) thyroidectomy, where the lack of tactile feedback limits tumor detection. The recent rise in robotic thyroid surgery further underscores the need for such a technique [30]. Reported mPSM rates in robotic procedures range from 2.9% to 10.5% [31]. In robotic thyroidectomy, magnified visualization facilitates identification of macroscopic margin involvement and enables additional resection to achieve clear margins. In contrast, lack of tactile feedback makes it more challenging to detect microscopic residual disease [32]. Analyses from the National Cancer Database have also shown a significantly higher incidence of mPSM with the robotic approach compared with conventional surgery, though surgeons tend to achieve similar margin outcomes [7,31,32,33,34]. The present technique could be beneficial across approaches, particularly in robotic thyroidectomy.

Our present findings also indicate that a sprayable fluorescent probe can be applied to identify margin involvement in more aggressive thyroid cancers such as ATC. ATC is one of the most lethal human malignancies, accounting for less than 2% of all thyroid cancers but nearly half of thyroid cancer-related deaths [35]. Positive surgical margins are common in ATC and are strongly correlated with local recurrence and poor prognosis. Median survival is typically under six months. We showed that PH10 spraying can successfully detect margin involvement in ATC, although the mean TBR was somewhat lower than that observed in PTC, likely due to tumor necrosis and dense fibrosis [36]. Achieving complete resection with the aid of PH10 may offer patients with ATC an opportunity for prolonged survival [37].

This study had several limitations. First, thyroid cancer cell lines often show features of highly aggressive thyroid cancer regardless of their original subtype [38]. Although we demonstrated margin detection with PH10 in both PTC and ATC, it remains unclear whether this approach is equally effective in more indolent thyroid cancer. Studies using patient-derived orthotopic xenograft (PDOX) models would help address this question. Second, the probe employed in the present study is not tumor biomarker-specific; instead, it targets hypoxic and mildly acidic tumor microenvironments (TMEs). This characteristic may improve generalizability and enable applications across multiple cancer types. However, the performance of a pH-responsive probe may differ under varying physiological conditions, including coexisting Hashimoto thyroiditis. Finally, because this is a preclinical study, additional validation is needed to establish its relevance and effectiveness in humans.

## 5. Conclusions

Topical spraying of PH10 provides rapid, tumor-specific fluorescence that labels orthotopic thyroid tumors in mouse models and identifies positive surgical margins after multiple-step FGS. This technique can improve intraoperative margin assessment, including during robotic thyroidectomy. These findings support the future translation of PH10-assisted FGS into clinical practice.

## Data Availability

Mouse experiment data supporting the findings of this study are available from the corresponding author upon reasonable request.

## Supplementary Materials

The following supporting information can be downloaded at: https://www.mdpi.com/article/10.3390/cancers18040632/s1, Figure S1: Chemical structure of PH10.

## Abbreviations

The following abbreviations are used in this manuscript:

mSPM	Microscopic positive surgical margin
FGS	Fluorescence-guided surgery
NIR	Near-infrared
PTC	Papillary thyroid carcinoma
ATC	Anaplastic thyroid carcinoma
ATCC	American Type Culture Collection
FBS	Fetal bovine serum
SOI	Surgical orthotopic implantation
PBS	Phosphate-buffered saline
CCK-8	Cell Counting Kit-8
OATP	Organic anion transporter peptide
BSP	Bromsulphthalein
BSA	Bovine serum albumin
DMSO	Dimethyl sulfoxide
mFI	Mean fluorescence intensities
TBR	Tumor-to-background ratio
H&E	Hematoxylin and eosin
CI	Confidence intervals
PDOX	Patient-derived orthotopic xenograft
TME	Tumor microenvironment
