# Supplementary figures and images for: A pH-Sensitive Sprayable Fluorescent Probe Enables Accurate Visualization of Thyroid Cancer Margins for Fluorescence-Guided Surgery in Orthotopic Mouse Models
Source: Cancers (Basel). 2026 Feb 15;18(4):632. doi: 10.3390/cancers18040632 (PMC12938921; doi:10.3390/cancers18040632)

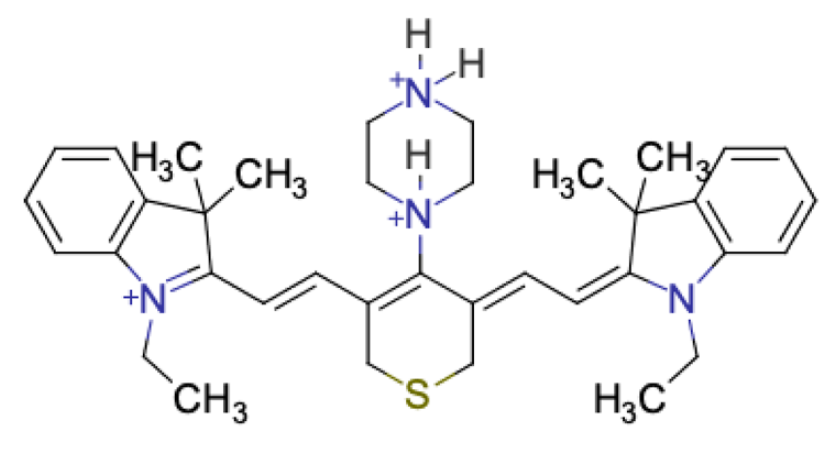

Supplement: Supplementary file 1 [file cancers-18-00632-s001.zip › Figure S1.png]
